# Mutation Detection in Activin A Receptor, Type I (*ACVR1*) Gene in Fibrodysplasia Ossificans Progressiva in An Iranian Family

**Published:** 2014-02-03

**Authors:** Ziba Morovvati, Saeid Morovvati, Gholamhossein Alishiri Alishiri, Seyed Hossein Moosavi, Reza Ranjbar, Yaser Bolouki Moghaddam

**Affiliations:** 1 Molecular Biology Research Center, Baqiyatallah University of Medical Sciences, Tehran, Iran; 2 Faculty of Medicine, Baqiyatallah University of Medical Sciences, Tehran, Iran; 3 School of Biology, Islamic Azad University Tehran Medical Branch, Tehran, Iran; 4 Student Research Center, Baqiyatallah University of Medical Sciences, Tehran, Iran

**Keywords:** FOP, c.617G>A Mutation, *ACVR1*

## Abstract

Fibrodysplasia Ossificans Progressiva (FOP, MIM 135100) is a rare genetic disease that is often inherited sporadically in an autosomal dominant pattern. The disease manifests in early life with malformed great toes and, its episodic and progressive bone formation in skeletal muscle after trauma is led to extra-articular ankylosis. In this study, a 17 year-old affected girl born to a father with chemical injury due to exposure to Mustard gas during the Iran-Iraq war, and her first degree relatives were examined to find the genetic cause of the disease. The mutation c.617G>A in the Activin A receptor, type I (*ACVR1*) gene was found in all previously reported patients with FOP. Therefore, peripheral blood samples were taken from the patient and her first-degree relatives. DNA was extracted and PCR amplification for *ACVR1* was performed. The sequencing of *ACVR1* showed the existence of the heterozygous c.617G>A mutation in the patient and the lack of it in her relatives. Normal result of genetic evaluation in relatives of the patient, ruled out the possibility of the mutation being inherited from parents. Therefore, the mutation causing disease in the child, whether is a new mutation with no relation to the father’s exposure to chemical gas, or in case of somatic mutation due to exposure to chemical gas, the mutant cells were created in father’s germ cells and were not detectable in his blood sample.

## Introduction

Fibrodysplasia Ossificans Progressiva (FOP, MIM 135100) is classified as a rare sporadic genetic disease that is inherited in an autosomal dominant pattern. The disease manifests in early life and, its episodic and progressive bone formation is led to extra-articular ankylosis of all major joints. Two clinical features define classic FOP: malformation of the great toes; and progressive heterotopic ossification (HO) in specific spatial patterns. Individuals with FOP appear normal at birth except for the characteristic malformations of the great toes which are present in all classically affected individuals (1). During the first decade of life, children with FOP develop painful and highly inflammatory soft tissue swellings (or flare-ups) that transform soft connective tissues, including aponeuroses, fascia, ligaments, tendons and skeletal muscles, into an armament-like encasement of bone (2-3). Most patients become confined to wheelchair by the third decade of their life and often succumb to pulmonary complications in their fifth or sixth decade of life. FOP has a prevalence of approximately 1 in 2 million worldwide, and shows no geographic, ethnic or gender preference. FOP becomes noticeable by great toe abnormalities (bone malformations), involving progressive ossification of skeletal muscle (heterotopic bone), fascia, tendons and ligaments. Any trauma to the muscles of a person with FOP such as invasive medical procedures and biopsies caused by intramuscular injections may trigger episodes of myositis followed by more rapid ossification. However, the disease, even in the absence of these factors, can happen in patients without previous warning. FOP must be distinguished from other genetic conditions of HO and nonhereditary (acquired) HO. Progressive osseous heteroplasia (POH) is a rare genetic condition of progressive HO defined clinically by cutaneous ossification that usually presents during childhood and progresses to involve subcutaneous and deep connective tissues, including muscle and fascia, in the absence of multiple features of Albright hereditary osteodystrophy (AHO) or hormone resistance (4).

FOP is differentiated from POH by congenital malformation of the great toes, preosseous inflammation or "flare-ups" and the lack of cutaneous ossification. Acquired HO commonly follows severe trauma, and can be observed at any age but is rare in young children (5). Acquired HO tends to occur at periarticular sites or at sites of blunt trauma or localized injury. FOP is commonly misdiagnosed as aggressive juvenile fibromatosis, lymphedema, or soft tissue sarcoma (6). Other diagnostic considerations are lymphoma, desmoids tumors, isolated congenital malformations, brachydactyly (isolated), and juvenile bunions. At present there is no definitive treatment or a way to stop disease progression. Genetic studies of the disease in various countries have reported the presence of c.617G>A mutation in *ACVR1* located on chromosome 2q24.1 in most cases of FOP patients (1, 7-9). This common mutation is located in exon 6 of *ACVR1* gene. The *ACVR1* gene encodes a type I bone morphogenic protein (BMP) transmembrane receptor which is involved in the BMP signaling pathway. Due to the low reproductive fitness of patients, most cases of FOP result from new mutations in this gene (10- 12).

## Case Report

Investigated in this study is a 17-year-old girl born in Ardebil (Iran) with a history of being hospitalized for four times. In her medical history, the girl was appeared normal at birth except for congenital malformations of the great toes. The patient’s parents were not related. At birth, the father was 34 and the mother 30 years old. Among the close relatives, there is no history of genetic disease. The patient was born by normal vaginal delivery with normal weight. Patient’s weight during growth was in minimal normal without high level physical activity.

**Fig 1:**
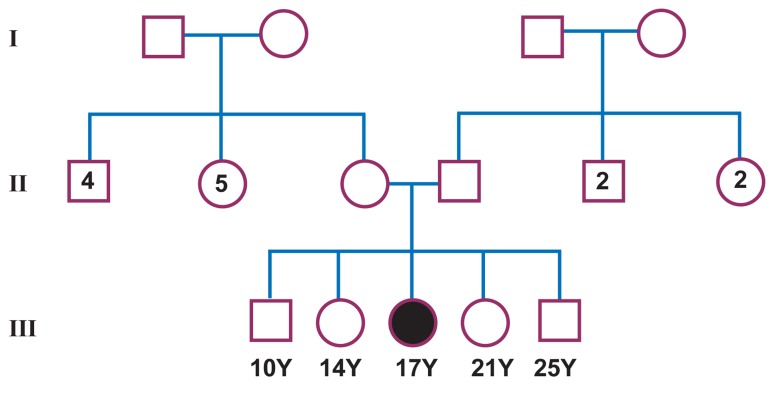
Pedigree of patient’s family.

**Fig 2:**
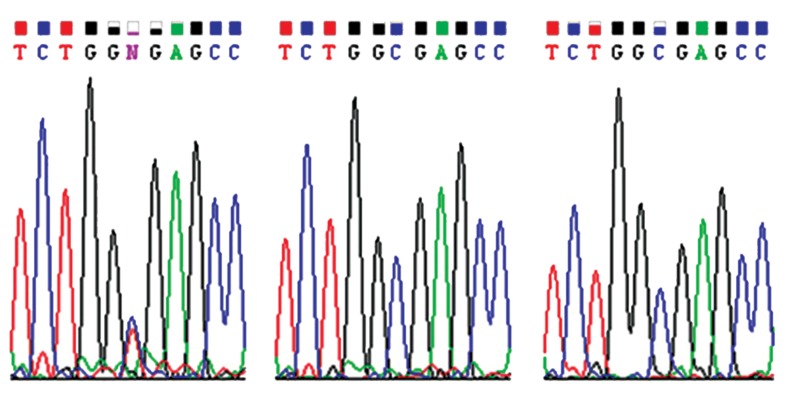
A. Presence of c.617G>A heterozygous mutation in the patient. B. Absence of c.617G>A mutation in the patient’s father. C. Absence of c.617G>A mutation in the patient’s mother.

The patient was first hospitalized at age 11 due to the initiation of depressomotor in shoulders, back, neck and left knee after trauma. Magnetic resonance imaging (MRI) of the right shoulder was normal during the hospitalization, but Tc-99 bone scan showed inflammatory response in the left shoulder and the right knee as well as some changes in the left shoulder. At this stage, muscle enzymes increased approximately 1.5 times higher than normal. Other hematologic, hormonal, and biochemical tests were normal. The patient was found to have FOP and she was treated with low dose ibuprofen and etidronate (200 mg) every alternate day. The patient was admitted for the second time in 16 years of age with more severe symptoms including right lower limb swelling and stiffness and pain in thigh and right leg. This time she had a severe scoliosis in her chest computed tomography scan (CT), hyper signal lesion in soft tissue of lower limb muscles in MRI, and bone formation in posterior thigh in xray of her lower limb. Three months later, the patient was referred and hospitalized with glenohumeral joint restrictions on both sides, right hip, knee and ankle, and left knee. Five months later at the age of 17, the patient referred again for the fourth time was hospitalized after complaining for a pain behind her right thigh and treated with medication and physiotherapy. In this study, the patient with FOP was examined for the genetic cause of her disease. After genetic counseling and assessing the familial pedigree (Fig 1), informed consent for genetic studies of all participants was obtained. Initially we investigated the c.617G>A mutation later found to be the common mutation causing the disease in the patient and her family. 5 ml of peripheral blood were collected from the patient, her parents, two sisters, and two brothers in tubes containing Ethylenediaminetetraacetic acid (EDTA). Leukocytes were separated from peripheral blood and DNA extraction was performed in accordance with standard phenol chloroform protocol (13). The fragment of *ACVR1* containing the c.617G>A mutation was amplified using PCR via the following primers FOP-F: 5′-CCA GTC CTT CTT CCT TCT TCC- 3′ and FOP-R: 5′-AGC AGA TTT TCC AAG TTC CAT C-3′. PCR conditions are as follows: 94˚C for 5 minutes, 30 cycles of 94˚C for 1 minute, 64˚C for 1 minute, and 72˚C for 1 minute followed by a final phase of 72˚C for 5 minutes. To determine the presence or absence of c.617G>A mutation in *ACVR1* gene, the PCR product was sequenced in both forward and reverse directions. Sequencing in the patient and relatives showed the presence of c.617G>A heterozygous mutation in the patient and absence in her immediate relatives. Figure 2A shows the presence of heterozygous mutation of c.617G>A in the patient. Respectively, figure 2B, C shows absence of the mentioned mutation in the patient’s father and mother. 

## Discussion

In 1993, Connor et al. (10) studied a five member family affected with FOP in three generations and discovered that the disease has an autosomal dominant inheritance pattern. In 1999, Lucotte et al. (14) sequenced the coding region of the Noggin gene in patients affected with FOP and identified a 42bp heterozygous deletion in its single exon region and attributed FOP occurrence to this mutation. In order to investigate the 42bp deletion of the reported noggin sequence, Xu et al. (15) examined 31 families with 1 or more FOP patients in 2000. However, they failed to detect the presence of the reported 42bp deletion. In the same year Feldman et al. (16) linked FOP to genes located in the 4q27-31 region but further studies have refuted the authenticity of the claim. Shore et al. (1) in 2006 attributed FOP to chromosome 2q23-24 using linkage analysis and identified an identical heterozygous mutation (c.617G>A) in all affected individuals of 7 families that were examined. The mutation located in the glycine-serine activation domain of *ACVR1* gene, which is a BMP type I receptor, and expressed in many tissues such as skeletal muscle and cartilage cells. In addition, Shore et al. (1) studied existence of the mutation in 32 sporadic cases with vague symptoms of disease and the presence of mutation in all patients was shown. In the same year, Lin et al. (7) reported the c.617G>A mutation in the *ACVR1* gene in a Taiwanese patient. A year later in 2007, Nakajima et al. (8) examined three Japanese patients with FOP for *ACVR1* mutations and identified the c.617G>A mutation in all three patients. In 2008, Kaplan et al. (9) evaluated 7 children with congenital malformations of the great toes. DNA sequence analysis found that all 7 of the children had the c.617G>A mutation. In 2008, Fukuda et al. (17), in addition to the former frequent c.617G>A mutation, identified a new c.1067G>A mutation in Japanese patients with FOP.

In this study, c.617G>A mutation in the *ACVR1* gene was examined in the patient and her family. The results showed that the patient was carrying the mutation but in her first-degree relatives, mutation was not found. Therefore, it appears that the mutation causing the disease in the daughter is either a new mutation that is not associated with the father’s exposure to chemical gas or in case of somatic mutation in father due to the exposure to chemical gas, the mentioned mutation has occurred in father’s germ cells which is not detectable in father’s blood sample. This article is the first report of genetic analysis of FOP in Iran and Middle Eastern countries in general.

## References

[1] Shore EM, Xu M, Feldman GJ, Fenstermacher DA, Cho TJ, Choi IH (2006). A recurrent mutation in the BMP type I receptor ACVR1 causes inherited and sporadic fibrodysplasia ossificans progressiva. Nat Genet.

[2] Cohen RB, Hahn GV, Tabas JA, Peeper J, Levitz CL, Sando A (1993). The natural history of heterotopic ossification in patients who have fibrodysplasia ossificans progressiva.A study of forty-four patients. J Bone Joint Surg Am.

[3] Rocke DM, Zasloff M, Peeper J, Cohen RB, Kaplan FS (1994). Age- and joint-specific risk of initial heterotopic ossification in patients who have fibrodysplasia ossificans progressiva. Clin Orthop Relat Res.

[4] Adegbite NS, Xu M, Kaplan FS, Shore EM, Pignolo RJ (2008). Diagnostic and mutational spectrum of progressive osseous heteroplasia (POH) and other forms of GNAS-based heterotopic ossification. Am J Med Genet A.

[5] Pignolo RJ, Foley KL (2005). Non-hereditary heterotopic ossification.Implications for injury, arthropathy, and aging. Clin Rev Bone Miner Metabol.

[6] Kitterman JA, Kantanie S, Rocke DM, Kaplan FS (2005). Iatrogenic harm caused by diagnostic errors in fibrodysplasia ossificans progressiva. Pediatrics.

[7] Lin GT, Chang HW, Liu CS, Huang PJ, Wang HC, Cheng YM (2006). De novo 617G-A nucleotide mutation in the ACVR1 gene in a Taiwanese patient with fibrodysplasia ossificans progressiva. J Hum Genet.

[8] Nakajima M, Haga N, Takikawa K, Manabe N, Nishimura G, Ikegawa S (2007). The ACVR1 617G>A mutation is also recurrent in three Japanese patients with fibrodysplasia ossificans progressiva. J Hum Genet.

[9] Kaplan FS, Xu M, Glaser DL, Collins F, Connor M, Kitterman J (2008). Early diagnosis of fibrodysplasia ossificans progressiva. Pediatrics.

[10] Connor JM, Skirton H, Lunt PW (1993). A three generation family with fibrodysplasia ossificans progressiva. J Med Genet.

[11] Pignolo RJ, Shore EM, Kaplan FS (2011). Fibrodysplasia ossificans progressiva: clinical and genetic aspects. Orphanet J Rare Dis.

[12] Shore EM, Glaser DL, Gannon FH (2000). Osteogenic induction in hereditary disorders of heterotopic ossification. Clin Orthop Relat Res.

[13] Sambrook J, Russell DW, Sambrook J, Russell DW (2001). Preparation and analysis of eukaryotic genomic DNA. Molecular cloning.

[14] Lucotte G, Sémonin O, Lutz P (1999). A de novo heterozygous deletion of 42 base-pairs in the noggin gene of a fibrodysplasia ossificans progressiva patient. Clin Genet.

[15] Xu MQ, Feldman G, Le Merrer M, Shugart YY, Glaser DL, Urtizberea JA (2000). Linkage exclusion and mutational analysis of the noggin gene in patients with fibrodysplasia ossificans progressiva (FOP). Clin Genet.

[16] Feldman G, Li M, Martin S, Urbanek M, Urtizberea JA, Fardeau M (2000). Fibrodysplasia ossificans progressiva, a heritable disorder of severe heterotopic ossification, maps to human chromosome 4q27-31. Am J Hum Genet.

[17] Fukuda T, Kanomata K, Nojima J, Kokabu S, Akita M, Ikebuchi K (2008). A unique mutation of ALK2, G356D, found in a patient with fibrodysplasia ossificans progressiva is a moderately activated BMP type I receptor. Biochem Biophys Res Commun.

